# 541. The Effect of Early Remdesivir Administration in COVID-19 Disease Progression

**DOI:** 10.1093/ofid/ofad500.610

**Published:** 2023-11-27

**Authors:** Moritz Platzer, David Totschnig, Christoph Wenisch, Alexander Zoufaly

**Affiliations:** Department for Infectious Diseases, Klinik Favoriten, Wiener Gesundheitsverbund, Wien, Wien, Austria; Department for Infectious Diseases, Klinik Favoriten, Wiener Gesundheitsverbund, Wien, Wien, Austria; Department for Infectious Diseases, Klinik Favoriten, Wiener Gesundheitsverbund, Wien, Wien, Austria; Department for Infectious Diseases, Klinik Favoriten, Wiener Gesundheitsverbund, Wien, Wien, Austria

## Abstract

**Background:**

Since the global outbreak of SARS-CoV-2, antiviral drugs have played a major role in the treatment of COVID-19 by reducing complications and mortality. Remdesivir is an effective therapeutic option for hospitalized patients with risk for clinical progression with COVID-19 who cannot receive nirmatrelvir/ritonavir mainly because of kidney & liver disease or issues with drug- interactions. Remdesivir can be administered for as long as 7 days after symptom onset according to treatment recommendations, but antiviral treatment work best when given as early as possible. In this study we investigated the impact of timing of remdesivir administration on clinical outcome.

**Methods:**

Retrospective clinical data from consecutive PCR-confirmed SARS-CoV-2 patients who received remdesivir and were hospitalized at the department of infectious diseases, Clinic Favoriten (Vienna) from the 1st of July 2021 until the 31st of April 2022 were analyzed. We divided patients into two groups of early (0-3 days since symptom onset) and late (≥ 4) remdesivir administration. The primary outcome was in hospital disease progression measured according to the WHO COVID-19 Clinical Progression Scale (≥1 point). In a multivariable logistic regression adjusted for age, sex, SARS-CoV2 variant, and COVID-19 vaccination status, clinical outcomes were assessed.

**Results:**

219 patients who received remdesivir could be included in the analysis. The ratio between early and late group was 67.6% to 32.4%. After adjusting for age, sex, vaccination status, and viral variant, patients who received remdesivir more than 4 days after symptom onset tended to have a higher probability of clinical deterioration (OR 2,13 95% CI 0,98 to 4,64, p= 0,056) including a higher probability for high-flow oxygen therapy (OR 2,52 95% CI 1,40 to 4,52, p= 0,002) or being admitted to ICU (OR 4,34 95% CI 1,38 to 13,67, p= 0,012). There was no significant difference in mortality.

**Table 1.**
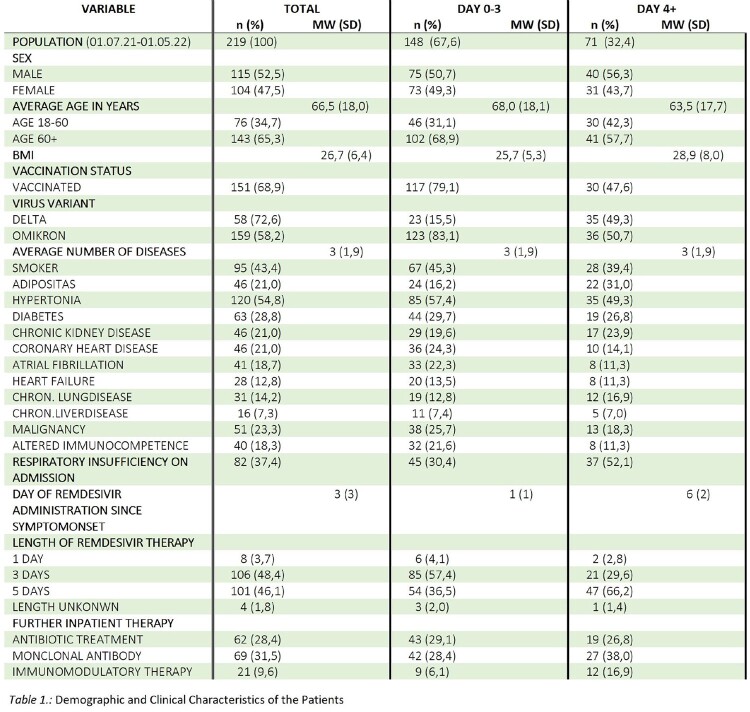
Demographic and Clinical Characteristics of the Patients

**Table 2.**
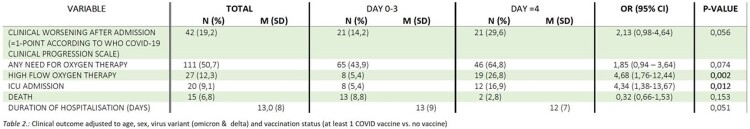
Clinical outcomes adjusted to age, sex, virus variant and vaccination status

**Conclusion:**

Our data show that the risk for complications of COVID19 including a need for high-flow oxygen therapy and ICU admission is elevated when remdesivir is administered after day 4 in hospitalized patients. This may indicate that patients at increased risk for severe disease who cannot take oral options should receive antiviral therapy with remdesivir as early as possible.

**Disclosures:**

**Moritz Platzer, MD**, Gilead: Grant/Research Support

